# Impact of Preoperative Continued Aspirin Therapy on Perioperative Bleeding Complications in Patients Undergoing Gastrectomy for Malignancy

**DOI:** 10.7759/cureus.65303

**Published:** 2024-07-24

**Authors:** Taisuke Matsuoka, Takahisa Fujikawa, Yuichiro Kawamura, Suguru Hasegawa

**Affiliations:** 1 Surgery, Kokura Memorial Hospital, Kitakyushu, JPN; 2 Gastroenterological Surgery, Fukuoka University Hospital, Fukuoka, JPN

**Keywords:** thromboembolic complications, bleeding complications, antithrombotic therapy, gastric cancer, continued aspirin therapy

## Abstract

Background

The question of whether antiplatelet therapy (APT) should be discontinued prior to gastrectomy is controversial. In this study, we investigated the impact of continuing aspirin preoperatively on perioperative bleeding and thromboembolic complications in patients receiving gastrectomy for malignancy.

Methods

The study cohort comprised 1001 patients with malignant gastric tumors who had undergone gastrectomy between 2005 and 2021. This study excludes emergency surgery. The patients were allocated to the following three groups: those who continued aspirin monotherapy prior to surgery (cAPT group), those who stopped receiving it seven days prior to surgery (dAPT group), and those who did not take APT at any stage (non-APT group). The differences between the groups in intraoperative and postoperative complications, such as bleeding and thromboembolism, were examined.

Results

The non-APT group comprised 682 patients, the dAPT group had 164, and the cAPT group had 155. There were 22 bleeding events (2.2%) in the whole cohort, 11 (1.1%) of which occurred in the non-APT group, six (3.7%) in the dAPT group, and five (3.2%) in the cAPT group. The differences between the three groups were not significant in terms of bleeding complications. There were 10 (1.0%) thromboembolic events in the whole cohort, five (0.7%) of which occurred in the non-APT group, four (2.4%) in the dAPT group, and one (0.6%) in the cAPT group. The differences between the three groups were not significant in terms of thromboembolic complications. In a multivariate analysis of the whole cohort, intraoperative blood loss (≥1000 mL) (p < 0.001, odds ratio (OR) = 11.8) and multidrug APT (p < 0.001, OR = 7.8) were both independent predictors of bleeding complications. However, continuing to take aspirin before surgery was not a risk factor for bleeding complications.

Conclusions

In patients with malignant gastric tumors, preoperative continuation of aspirin monotherapy has no impact on either intraoperative or postoperative bleeding. Gastrectomy can be performed safely, even in patients who continue aspirin treatment.

## Introduction

Cardiovascular and cerebrovascular diseases have become more common in recent years because of the aging of populations. Antithrombotic therapy (ATT), which includes antiplatelet therapy (APT) and anticoagulant therapy (ACT), prevents secondary cardiovascular or cerebrovascular diseases. Discontinuation of ATT is generally considered to reduce the risk of perioperative bleeding complications. However, such withdrawal may increase the risk of thromboembolic complications [1,2]. Considering the balance between hemorrhagic complications and infarction complications, some literature recommends continuing APT preoperatively and shows its safety [3,4]. However, the POISE-2 (PeriOperative ISchemic Evaluation-2) trial showed that continued use of aspirin increases the risk of significant bleeding while having no effect on the rates of mortality or nonfatal myocardial infarction [5]. Despite the fact that this topic has been increasingly discussed and a number of strategies have been suggested, the best way to manage ATT throughout the perioperative period remains controversial [6-8].

According to the Global Cancer Observatory (GLOBOCAN), gastric cancer is the fifth most common cancer in the world, accounting for approximately one million cases and an estimated 660,000 deaths [9]. Gastric cancer is typically treated with a gastrectomy combined with lymph node dissection; the complications and death rates related to this procedure are estimated to be 20-40% and 1-5%, respectively [10,11]. Controlling bleeding during surgery can guarantee a safe field of vision, and controlling thromboembolic complications after surgery may reduce the rate of death following surgery.

In 2009, we drew on several guidelines to develop an antithrombotic management protocol (the Kokura Protocol) for patients on ATT. This protocol specifies that patients with thromboembolic risks should continue aspirin monotherapy until the day prior to surgery. In this study, we investigated whether continuation of preoperative aspirin affects perioperative bleeding and thromboembolism in patients undergoing gastrectomy for malignancy using our protocol.

## Materials and methods

**Figure 1:**
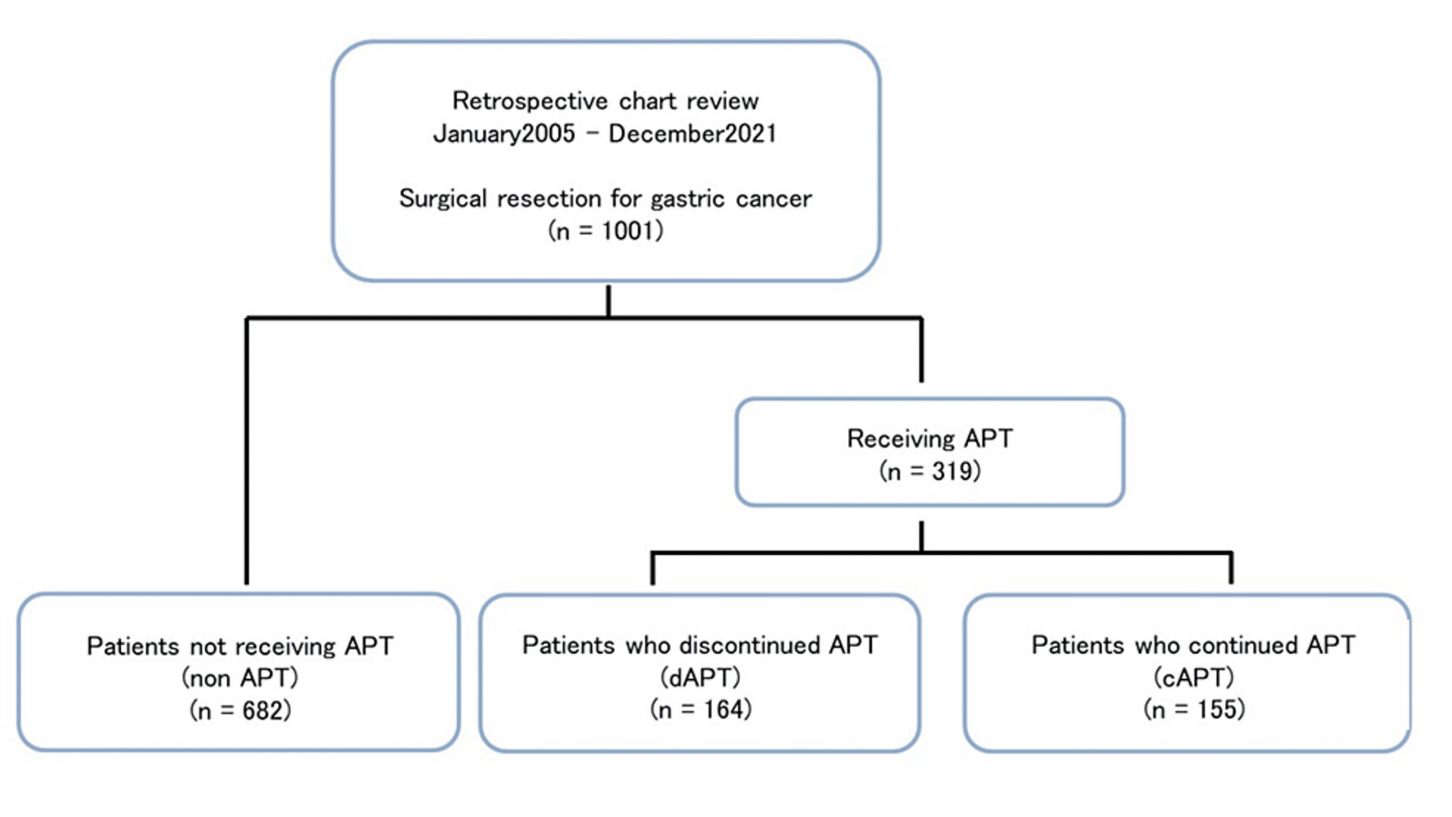
Flowchart of this study A total of 1001 patients who had undergone gastrectomy for malignant gastric tumors were divided into three groups according to perioperative antiplatelet therapy status. APT: antiplatelet therapy; dAPT: discontinued antiplatelet therapy; cAPT: continued antiplatelet therapy

This single-center retrospective cohort study included 1001 patients who had undergone gastrectomy for malignant gastric tumors between January 2005 and December 2021 in our institution. The patients were divided into three groups according to their preoperative APT status: 155 who had received continued aspirin monotherapy preoperatively (cAPT group), 164 who had discontinued APT seven days before surgery (dAPT group), and 682 who had not received APT at any stage (non-APT group). Before the Kokura protocol was established, antiplatelet agents were rested for seven days before surgery, so patients using APT were assigned to the dAPT group. After the protocol was established, aspirin was continued until the day before surgery, so patients using APT were assigned to the cAPT group. Randomization was not performed, but as this was in accordance with the protocol, there was no arbitrary selection by surgeons. Fifteen cases were seen in this investigation using two antiplatelet medications and were defined as dual antiplatelet therapy (DAPT). Of the two oral antiplatelet drugs, one was discontinued one week before surgery, and only aspirin was continued, and the patient was included in the cAPT group. Anesthesia for surgery was general anesthesia, and epidural anesthesia was not used in ATT cases. Among non-ATT cases, only patients who requested epidural anesthesia were given, and the catheter was removed three days after surgery (Figure 1).

Intraoperative and postoperative complications, including bleeding and thromboembolic complications, were compared between these groups. The Clavien-Dindo classification (CDC) was used to evaluate postoperative complications, with CDC class II or higher being deemed significant. Bleeding complications included gastrointestinal, intra-abdominal, and abdominal wall bleeding. Thromboembolic complications included myocardial and cerebral infarctions. Morbidity and mortality were defined as occurring within 30 days after surgery.

CHADS-2 scores, which are cumulative and based on six clinical variables, namely age greater than or equal to 75 years, diabetes mellitus (DM), congestive heart failure, hypertension, and a history of stroke or transient ischemic attack, were used to assess and categorize the patients’ postoperative thromboembolic risk [12,13]. This study was approved by Kokura Memorial Hospital’s Institutional Review Board (no. 19061903).

Perioperative antithrombotic protocol

**Figure 2:**
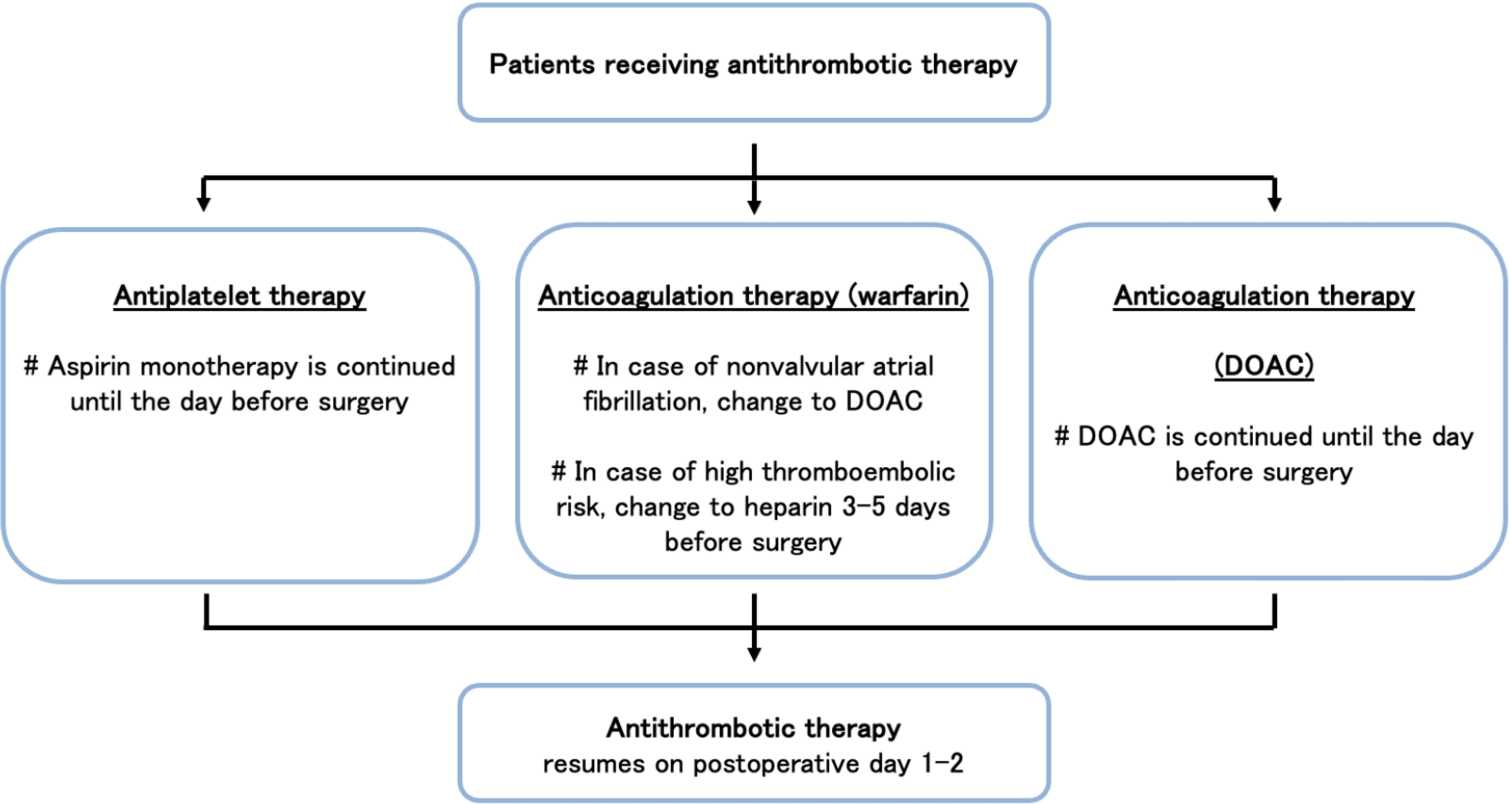
Institutional protocol (“Kokura Protocol”) for perioperative antithrombotic therapy (antiplatelet and anticoagulation therapy) DOAC: direct oral anticoagulant This figure was reprinted and adapted with permission [8].

In 2009, we established a protocol for perioperative antithrombotic management (Kokura protocol) in which APT in the form of aspirin monotherapy is continued until the day before surgery [1]. In patients taking multiple APT agents (such as aspirin plus P2Y12 inhibitor), the other types of APT are withdrawn seven days before surgery, whereas aspirin monotherapy is continued. Aspirin is restarted early postoperatively (within two days) after an adequate assessment of hemostasis. In a patient with nonvalvular atrial fibrillation, a direct oral anticoagulant or bridging heparin is substituted for ACT five days before surgery [2]. Figure 2 shows the flowchart for the Kokura protocol.

Statistical analysis

Data were collected and analyzed using JMP Pro software (version 11.0.0; SAS Institute, Cary, NC, USA). Data are shown as frequency and proportion (%) and the means ± standard error of the mean. χ2 tests, Fischer’s exact test, and one-way analysis of variances (ANOVA) were performed to compare data between the three groups as appropriate. Differences between the three groups were assessed by one-way ANOVA. When one-way ANOVA was applied, the Tukey-Kramer honestly significant difference (HSD) test was used to identify significant differences between groups. Univariate and multivariate logistic regression analyses were used to determine risk factors influencing postoperative bleeding and thromboembolic complications. Only variables with p<0.05 in the univariate analysis were considered significant and included in the subsequent multivariate analysis. Significant differences were defined as p<0.05.

## Results

Clinical characteristics of patients

A total of 1001 patients underwent gastrectomy for malignant gastric tumors during the study period. Table 1 shows these patients’ background characteristics according to the study groups. At the time of diagnosis of gastric cancer, 319 patients (31.9% of the full cohort) had been receiving APT, whereas 682 (68.1%) patients had not received APT at any stage (non-APT group), 164 (16.4%) had discontinued antithrombotic drugs seven days before surgery (dAPT group) and 155 (15.5%) had continued aspirin until one day before surgery (cAPT group). The three groups differed significantly in age, sex ratio, severity assessment scale (SAS), histories of hypertension and DM, and CHADS-2 scores. Post-hoc tests revealed a significant difference between the non-APT and both APT groups (dAPT and cAPT groups). In contrast, the post-hoc tests did not reveal any significant differences between the dAPT and cAPT groups in age, sex ratio, ASA, or CHADS-2 score.

**Table 1:** Demographics and clinical features of patients ^* ^one-way analysis of variance (ANOVA); ^# ^Fisher's exact test; BMI: body mass index; ASA-PS: American Society of Anesthesiologists Physical Status; HTN: hypertension; APT: antiplatelet therapy; cAPT: continued aspirin monotherapy prior to surgery; dAPT: discontinued aspirin monotherapy seven days prior to surgery

Variable	Total (n = 1001)	Non-APT (n = 682)	dAPT (n = 164)	cAPT (n = 155)	p-value
Age mean ± SD	70.8 ± 10.9	68.9 ± 11.5	75.0 ± 8.0	75.2 ± 7.6	<0.001*
Male gender, n (%)	733 (73.2)	459 (67.3)	141 (86.0)	133 (85.8)	<0.001^#^
BMI <30kg/m^2^, n (%)	983 (98.2)	668 (97.8)	161 (98.1)	154 (97.4)	0.58^#^
Performance state <2, n (%)	979 (98.2)	668 (97.9)	160 (97.6)	151 (97.4)	0.85^#^
ASA-PS <2, n (%)	756 (75.5)	592 (86.3)	89 (54.2)	75 (48.4)	<0.001^#^
HTN, n (%)	367 (36.6)	225 (33.0)	63 (38.4)	79 (51.0)	<0.001^#^
Diabetes mellitus, n (%)	190 (19.0)	94 (13.8)	40 (24.4)	56 (36.1)	<0.001^#^
CHADS-2 score >1, n (%)	692 (69.1)	407 (59.7)	147 (89.6)	138 (89.0)	<0.001^#^

Perioperative characteristics and postoperative morbidity

Table 2 provides an overview of the study group-specific perioperative characteristics and postoperative morbidity. Open surgery was performed on 53.1% of the non-APT group, 84.0% of the dAPT group, and 38.1% of the cAPT group. These differences are significant (p < 0.001). The duration of surgery was 279 ± 103.5 min in the non-APT group, 255 ± 74.6 min in the dAPT group, and 288 ± 104 min in the cAPT group, being significantly longer in the cAPT group on post-hoc testing (p = 0.007). Blood loss was 207 ± 361 mL in the non-APT group, 244 ± 309 mL in the dAPT group, and 122 ± 260 mL in the cAPT group, being significantly less in the cAPT group on post-hoc testing (p = 0.003). Bleeding complications of CDC 2 or higher occurred in 1.6% of the non-APT group, 3.7% of the dAPT group, and 3.2% of the cAPT group; these differences are not significant (p = 0.138). Thromboembolic complications of CDC 2 or higher occurred in 0.7% of the non-APT group, 2.4% of the dAPT group, and 0.6% of the cAPT group; these differences were not significant (p = 0.134).

**Table 2:** Perioperative characteristics and postoperative morbidity ^* ^one-way analysis of variance (ANOVA); ^# ^Fisher's exact test; APT: antiplatelet therapy; cAPT: continued aspirin monotherapy prior to surgery; dAPT: discontinued aspirin monotherapy seven days prior to surgery

Characteristics	Non-APT (n = 682)	dAPT (n = 164)	cAPT (n = 155)	p-value
Duration of surgery (min) mean ± SD	279 ± 103.5	255 ± 74.6	288 ± 104.0	0.007*
Open surgery, n (%)	362 (53.1%)	138 (84.1%)	59 (38.1%)	<0.001^#^
Laparoscopic or robotic surgery, n (%)	320 (46.9%)	26 (15.9%)	96 (61.9%)	<0.001^#^
Blood loss (mL) mean ± SD	207 ± 361	244 ± 309	122 ± 260	0.003*
Postoperative morbidity ≥ Grade 2, n (%)	92 (13.5%)	40 (24.4%)	26 (16.8%)	0.004^#^
Postoperative morbidity ≥ Grade 3, n (%)	36 (5.3%)	20 (12.2%)	15 (9.7%)	0.003^#^
Bleeding complications ≥ Grade 2, n (%)	11 (1.6%)	6 (3.7%)	5 (3.2%)	0.138^#^
Bleeding complications ≥ Grade 3, n (%)	11 (1.6%)	3 (1.9%)	4 (2.5%)	0.625^#^
Thromboembolism complications ≥ Grade 2, n (%)	5 (0.7%)	4 (2.4%)	1 (0.6%)	0.134^#^
Thromboembolism complications ≥ Grade 3, n (%)	5 (0.7%)	4 (2.4%)	1 (0.6%)	0.134^#^

Postoperative bleeding and thromboembolic complications

We investigated the whole cohort’s postoperative bleeding complications in more detail. Bleeding complications occurred in 22 patients overall. The most frequent sites of bleeding were anastomotic and intraperitoneal. There were 11 cases (1.6%) in the non-APT group, six (3.7%) in the dAPT group, and five (3.2%) in the cAPT group; these differences are not significant. In surgery for gastric cancer, we basically perform routine lymph node dissections. Unless there is a special reason, we do not perform cholecystectomy or splenectomy. Two of the patients who had bleeding complications underwent cholecystectomy. Both cases were gastrointestinal bleeding and cholecystectomy was not regarded to be causally related to gastrointestinal bleeding. There were no mortality-related bleeding complications. We also investigated the whole cohort’s postoperative thromboembolic complications in more detail. These occurred in 10 patients overall. The most frequent types of thromboembolism were cerebral and myocardial infarctions. There were five cases (0.7%) in the non-APT group, four (2.4%) in the dAPT group, and one (0.6%) in the cAPT group; these differences are not significant. There were five mortality-related thromboembolic complications.

Tables 3-4 present the results of univariate and multivariate analyses pertaining to bleeding and thromboembolic complications in the whole cohort. Risk factors for bleeding and thromboembolic complications were extracted based on previous papers [8]. Regarding bleeding complications, univariate analysis revealed use of ACT, multidrug ATT, multidrug APT, and high intraoperative blood loss (≥1000 mL) were significant risk factors for bleeding complications. In the multivariable analysis, multidrug APT (p < 0.001, odds ratio (OR) = 7.8) and high blood loss during surgery (≤1000 mL) (p < 0.001, OR = 11.8) were found to be significant, but continued aspirin therapy before surgery was not. The univariable analyses revealed that age (≥75), performance status (PS) (≥2), and history of DM were linked to thromboembolic complications, although the multivariable analysis indicated that either PS, age (≥75), or history of DM was not significantly related to thromboembolism.

**Table 3:** Analysis of univariate data for bleeding and thromboembolic events following gastric surgery (n = 1001) The number and percentage of patients are displayed as the results. PS: physical status; CABG: coronary artery bypass grafting; PCI: percutaneous coronary intervention; DM: diabetes mellitus; HTN: hypertension; HIBL: high intraoperative blood loss; ACT: anticoagulant therapy; APT: antiplatelet therapy; ATT: antithrombotic therapy

Type of complication		Bleeding complications		Thromboembolic complications	
Variable	n	Present(%)	Univariate p	Present(%)	Univariate p
Age <75, n (%)	572	13 (2.3)	1	2 (0.3)	0.023
Male gender, n (%)	733	18 (2.5)	0.469	9 (1.2)	0.304
PS ≥ 2, n (%)	93	3 (3.2)	0.45	4 (4.3)	0.009
CHADS-2 ≥ 1, n (%)	673	19 (2.8)	0.101	9 (1.3)	0.188
History of CABG, n (%)	50	3 (6.0)	0.092	1 (2.0)	0.402
History of PCI, n (%)	174	5 (2.9)	0.566	2 (1.1)	0.688
History of PCI or CABG, n (%)	207	7 (3.4)	0.189	3 (1.4)	0.44
History of DM, n (%)	190	3 (1.6)	0.783	5 (2.6)	0.026
History of HTN, n (%)	367	11 (3.0)	0.262	6 (1.6)	1
ACT used, n (%)	101	6 (5.9)	0.002	3 (3.0)	0.071
APT used, n (%)	319	11 (3.4)	0.102	5 (1.6)	0.304
Multiple ATT used, n (%)	49	4 (8.2)	0.019	0 (0)	1
Multiple APT used, n (%)	64	7 (10.9)	<0.001	0 (0)	1
Continuation of APT, n (%)	155	5 (3.2)	0.367	1 (0.6)	1
HIBL ≥ 1000 mL, n (%)	29	4 (13.8)	0.003	1 (3.4)	0.256
Lap or Robot, n (%)	442	12 (2.7)	0.387	3 (0.7)	0.526

**Table 4:** Analysis of multivariate data for bleeding and thromboembolic events following gastric surgery (n = 1001) OR: odds ratio; CI: confidence interval; PS: performance status; ACT: anticoagulant therapy; APT: antiplatelet therapy; ATT: antithrombotic therapy; HIBL: high intraoperative blood loss; DM: diabetes mellitus

Type of complication	Bleeding complications			Thromboembolic complications		
Variable	Multivariate p	OR	95% CI	Multivariate p	OR	95% CI
ACT used	0.25	2.5	00.5-12.2	-	-	-
Multiple ATT used	0.66	1.6	0.2-10.6	-	-	-
Multiple APT used	<0.001	7.8	2.8-21.6	-	-	-
HIBL ≥ 1000 mL	<0.001	11.8	3.5-40.1	-	-	-
Age ≥ 75	-	-	-	0.06	1.1	1.0-1.2
PS ≥ 2	-	-	-	0.11	3.1	0.8-12.6
History of DM	-	-	-	0.06	3.5	1.0-12.4

## Discussion

In this study, we investigated bleeding and thromboembolic complications associated with surgery for malignant gastric tumors and the safety of continuing aspirin. We found no significant differences in the incidence of bleeding complications between the study groups when aspirin was continued throughout the perioperative period. Multivariate analysis showed that high intraoperative blood loss (≥1000 mL) and multiple APTs were significant risk factors for bleeding complications, and continuing preoperative aspirin therapy was not a risk factor for bleeding complications.

Thromboembolic complications are often severe, leading to postoperative morbidity and mortality [5,14]. Similarly, our study demonstrated that thromboembolic complications were CDC 4-5. On the other hand, bleeding complications were CDC 2-4 in this study. There were no deaths related to bleeding complications. The mortality of bleeding complications can be reduced by appropriate treatments such as blood transfusions, endoscopy, and surgery [15,16]. Given that thromboembolic complications are more likely to be severe than bleeding complications, the focus should be on preventing the former.

The European Society of Cardiology and the European Society of Anaesthesiology Guidelines on non-cardiac surgery suggest continuing low-dose aspirin during the perioperative period, provided the risk of bleeding is not particularly severe [3]. We have previously shown that perioperative cAPT is safe for different types of abdominal surgery [8,17,18]. Aoyama et al. have reported that gastrectomy can be safely performed in gastric cancer patients receiving antithrombotic or anticoagulant drugs [19], but during the perioperative phase, care must be used while giving multi-antithrombotic medications.

DAPT, including aspirin plus a P2Y12 inhibitor, is essential for the management of acute coronary syndrome in the re-endothelialization phase following percutaneous coronary intervention (PCI). Continuing DAPT perioperatively may increase the risk of bleeding complications. A meta-analysis revealed that in patients with acute coronary syndromes and those undergoing PCI, the administration of both clopidogrel and aspirin decreased the OR of the composite outcome of mortality, reinfarction, and stroke. However, receiving dual therapy was linked to a noticeably higher risk of serious hemorrhage [20,21]. Our study also showed that multiple APTs were significant risk factors for bleeding complications. We think that in order to strike a healthy balance between bleeding and thromboembolism consequences, aspirin alone may be more appropriate than DAPT.

This study’s limitations were that it was a retrospective, single-institutional study and included patients with widely varying characteristics. The duration of this study was relatively long, with background characteristics varying during that period. The ratio of open surgery was high at the beginning of the study, after which the ratios of laparoscopic and robotic surgery increased progressively. There was more open surgery in the dATP group as a result of this change, at the same time as the transfer from open to laparoscopic surgery. Also, guidelines for and management of perioperative antithrombotic treatment have gradually changed. Thus, the cAPT group was more likely to have undergone laparoscopic and robotic surgery, with an associated longer operation time and a smaller amount of blood loss. This restriction will be mitigated in a prospective multi-institutional study or in further follow-up investigations.

## Conclusions

No increase was found in the incidence of bleeding complications when aspirin was continued preoperatively. Multivariate analysis showed continuing preoperative aspirin therapy was not a risk factor for bleeding complications. Thus, gastrectomy for malignancy can be performed safely, even in patients who are receiving ongoing aspirin therapy.
